# Static and Free-Boundary Vibration Analysis of Egg-Crate Honeycomb Core Sandwich Panels Using the VAM-Based Equivalent Model

**DOI:** 10.3390/ma18174014

**Published:** 2025-08-27

**Authors:** Ruihao Li, Hui Yuan, Zhenxuan Cai, Zhitong Liu, Yifeng Zhong, Yuxin Tang

**Affiliations:** 1 School of Civil Engineering, Chongqing University, Chongqing 400045, China; ruihaoli2001@163.com (R.L.); 202216021133t@stu.cqu.edu.cn (H.Y.); 20223291@stu.cqu.edu.cn (Z.C.); 20223449@stu.cqu.edu.cn (Z.L.); 15823179624@163.com (Y.T.); 2 Key Laboratory of New Technology for Construction of Cities in Mountain Area, Chongqing University, Chongqing 400045, China

**Keywords:** egg-crate honeycomb core, sandwich panel, variational asymptotic method, equivalent plate model, buckling analysis, local field reconstruction

## Abstract

This study proposes a novel egg-crate honeycomb core sandwich panel (SP-EHC) that combines the structural advantages of conventional lattice and grid configurations while mitigating their limitations in stability and mechanical performance. The design employs chamfered intersecting grid walls to create a semi-enclosed honeycomb architecture, enhancing out-of-plane stiffness and buckling resistance and enabling ventilation and drainage. To facilitate efficient and accurate structural analysis, a two-dimensional equivalent plate model (2D-EPM) is developed using the variational asymptotic method (VAM). This model significantly reduces the complexity of three-dimensional elasticity problems while preserving essential microstructural characteristics. A Reissner–Mindlin-type formulation is derived, enabling local field reconstruction for detailed stress and displacement evaluation. Model validation is conducted through experimental testing and three-dimensional finite element simulations. The 2D-EPM demonstrates high accuracy, with static analysis errors in load–displacement response within 10% and a maximum modal frequency error of 10.23% in dynamic analysis. The buckling and bending analyses, with or without initial deformation, show strong agreement with the 3D-FEM results, with deviations in the critical buckling load not exceeding 5.23%. Local field reconstruction achieves stress and displacement prediction errors below 2.7%, confirming the model’s fidelity at both global and local scales. Overall, the VAM-based 2D-EPM provides a robust and computationally efficient framework for the structural analysis and optimization of advanced sandwich panels.

## 1. Introduction

Sandwich panels have become indispensable in modern engineering due to their exceptional combination of high stiffness, strength, and low weight [1,2]. A typical sandwich structure consists of two thin, stiff face sheets bonded to a lightweight core, which can occupy more than half the volume and weight of the panel [3,4]. The core thus plays a critical role in mechanical performance, and extensive research has been devoted to optimizing core configurations for improved strength-to-weight ratios [5,6]. Early studies focused on honeycomb cores—named for their biomimetic hexagonal cells—which endow panels with high out-of-plane compression and shear stiffness at minimal weight [7,8].

Recently, numerous analytical models have been developed to characterize sandwich panels, ranging from classical first-order shear deformation theory [9,10] to higher-order and layer-wise (zig-zag) theories [11,12]. These models improve accuracy in predicting bending and vibration by accounting for transverse shear and layer discontinuities, but they also introduce complexity (e.g., requiring additional degrees of freedom for each layer) and may not easily accommodate complex core architectures. As a result, the analysis and design of novel core geometries often rely on numerical methods. Finite element analysis (FEA) is frequently used to evaluate sandwich panel behavior, yet full three-dimensional (3D) modeling of a sandwich structure with intricate cellular cores is computationally expensive and time-consuming [13,14].

To alleviate this issue, homogenization and equivalent single-layer approaches have been explored, wherein a periodic unit cell of the core is analyzed to derive effective properties for an equivalent continuum panel [15,16]. Such micromechanical approaches greatly reduce computational cost by replacing the detailed core with a homogeneous plate model [17,18]. However, standard homogenization can lose microstructural detail and may sacrifice accuracy in predicting local stress concentrations or higher-order effects, especially for advanced core designs [19]. This limitation motivates the pursuit of more refined yet efficient modeling techniques.

In parallel with advances in modeling, a rich variety of innovative core designs has emerged to overcome the limitations of conventional honeycombs and lattice grids [20,21]. Researchers have proposed architected cellular materials and metamaterial cores that achieve superior mechanical performance through geometry [22,23]. For example, auxetic re-entrant honeycombs (with a negative Poisson’s ratio) have demonstrated enhanced energy absorption and stiffness compared to regular hexagonal cells [24,25]. Novel re-entrant chiral and hybrid honeycomb patterns have been shown to improve impact resistance and specific energy absorption under compression [26,27]. Elliptical and other non-periodic cell geometries have likewise been tailored to enhance in-plane stiffness or to achieve desirable anisotropic properties [28]. Beyond honeycombs, lattice and grid-like cores manufactured via advanced methods (e.g., additive manufacturing) are gaining attention [29,30].

Periodic truss lattices and orthogrid structures can be optimized to align with load paths, and they offer the promise of multi-functionality—for instance, open-cell lattice cores can double as heat exchange surfaces for thermal management [31,32]. Despite these advantages, conventional grid and lattice sandwich structures face several limitations. First, purely open lattice cores are prone to local instabilities: under concentrated or transverse loads, their slender struts can lead to highly localized stress and early failure in the core [33,34]. This makes lattice cores less effective in carrying impact loads without additional reinforcement.

Second, while grid-stiffened panels (e.g., orthogrid or iso-grid structures) provide high in-plane stiffness, they often exhibit complex buckling modes and stress concentrations at nodal intersections or cut-outs. Orthogonal grid patterns increase stiffness but may require curved or hierarchical modifications to delay buckling in critical regions [35]. In general, fully exploiting these novel core architectures is challenging because their mechanical behavior is not as well understood as that of traditional honeycombs. Designers lack simple formulas or design guidelines and instead must resort to extensive FEA for each new topology [36,37]. This trial-and-error approach is costly and time-consuming, underscoring the need for efficient analytical or equivalent models to guide the design of advanced sandwich cores.

**Figure 1:**
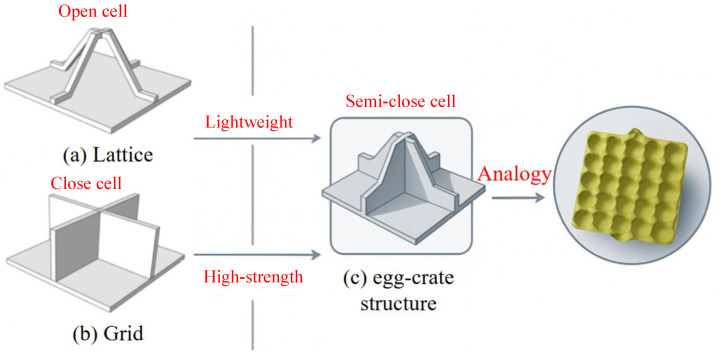
Comparison of (**b**) grid, (**a**) lattice, and (**c**) 3D egg-crate structures.

The present work introduces a novel “egg-crate” honeycomb core sandwich panel (SP-EHC), along with a high-fidelity yet computationally efficient modeling framework for its static and dynamic analysis. The core concept of SP-EHC is derived from conventional lattice and grid structures, aiming to integrate their strengths while mitigating weaknesses. The core consists of an orthogonal grid network with intersecting walls, but unlike a simple rectangular grid, the intersection regions are chamfered to form a semi-enclosed cell geometry as shown in Figure 1. This egg-crate-like configuration creates a pattern of contiguous cells analogous to a honeycomb, but with open corners and pathways. The chamfered grid walls serve to enhance structural strength and buckling resistance by eliminating sharp corner stress concentrations and providing smoother load transfer between intersecting ribs. Meanwhile, the semi-enclosed cells preserve the panel’s lightweight characteristics and introduce practical benefits such as ventilation and drainage.

To rigorously analyze the SP-EHC design, the two-dimensional equivalent plate model (2D-EPM) using the variational asymptotic method (VAM) is developed. The VAM, originally formulated by Cesnik and Hodges for composite structures [38,39], is a multi-scale asymptotic technique that reduces a 3D elasticity problem to a lower-dimensional model without ad hoc assumptions. The core idea of the VAM is to exploit the small geometric parameter (the core thickness-to-panel length ratio, in this case) and systematically minimize the 3D strain energy functional to obtain a simplified model that still captures the essential physics [40]. In practice, this involves decomposing the displacement field into a 2D (mid-plane) part plus a warping field, and deriving effective plate stiffness by averaging the 3D behavior over a representative unit cell. Unlike classical plate theories, the VAM-based approach inherently accounts for the actual microstructure of the core, enabling accurate prediction of both global responses and local fields.

This method has been successfully applied to periodic lattice and honeycomb structures in recent studies, showing that a VAM-derived plate model can reproduce the full 3D behavior with reduced computational cost [41,42]. For example, Wang et al. [43] used a VAM-based equivalent model for triangular honeycomb panels and achieved excellent agreement with experimental and 3D FEA results (for bending deflections, buckling loads, and vibration frequencies) while improving computation efficiency by orders of magnitude. In this study, the variational asymptotic method (VAM) is employed to derive the three-dimensional strain energy of the SP-EHC cell, followed by an asymptotic expansion to extract the governing two-dimensional plate equations and construct the equivalent stiffness matrices. Based on the 2D solution, recovery relations are established to reconstruct local stress and strain fields within the unit cell. A key advantage of the VAM framework lies in its multi-scale capability: it enables efficient computation of global deflections and stresses while also allowing for detailed reconstruction of micro-scale stress distributions—such as those in cell walls—for failure and fatigue analysis. This level of resolution is not readily achievable through conventional homogenization techniques.

## 2. VAM-Based Equivalent Model of SP-EHC

### 2.1. Three-Dimensional Energy Expression of SP-EHC

From the perspective of the equivalent single-layer theory, the 3D displacement field $u_{i}$ of SP-EHC can be expressed using the 2D displacement field ${\bar{u}}_{i}$ and the warping functions $\chi_{i}$ as

(1) $$\begin{array}{cc} u_{1}\left(x_{\alpha },y_{i}\right) & =\underline{{\bar{u}}_{1}\left(x_{\alpha }\right)-\langle \xi y_{3}\rangle {\bar{u}}_{3,1}\left(x_{\alpha }\right)}+\chi_{1}\left(x_{\alpha };y_{i}\right)\\ u_{2}\left(x_{\alpha },y_{i}\right) & =\underline{{\bar{u}}_{2}\left(x_{\alpha }\right)-\langle \xi y_{3}\rangle {\bar{u}}_{3,2}\left(x_{\alpha }\right)}+\chi_{2}\left(x_{\alpha };y_{i}\right)\\ u_{3}\left(x_{\alpha },y_{i}\right) & =\underline{{\bar{u}}_{3}\left(x_{\alpha }\right)}+\chi_{3}\left(x_{\alpha };y_{i}\right) \end{array}$$

where ξ denotes the small parameter representing the ratio of the micro- to macro-scales, the angle brackets indicate integration over the unit cell volume (Ω), and the underlined terms represent the displacements according to classical plate theory.

The explicit form of $u_{i}$ can be derived from Equation (1) by applying a volume-averaging procedure over the representative unit cell:

(2) $${\bar{u}}_{1}=\langle u_{1}\rangle +\langle \xi y_{3}\rangle {\bar{u}}_{3,1},\;{\bar{u}}_{2}=\langle u_{2}\rangle +\langle \xi y_{3}\rangle {\bar{u}}_{3,2},\;{\bar{u}}_{3}=\langle u_{3}\rangle$$

Considering that the local coordinate system originates from an arbitrary point within the unit cell, the 3D displacement field can be projected to obtain an equivalent 2D displacement field using Equation (2). To ensure consistency with the macroscopic kinematics, the warping functions are subject to the following constraint:

(3) $$\int_{\Omega }\chi_{i}\;d\Omega =\langle \chi_{i}\rangle =0$$

By introducing the concept of strain decomposition with local rotation, the 3D strain field accounting for small rotations can be expressed as follows:

(4) $$\varepsilon_{ij}=\frac{1}{2}\left(\frac{\partial u_{i}}{\partial x_{j}}+\frac{\partial u_{j}}{\partial x_{i}}\right)-\delta_{ij},$$

where $\delta_{ij}$ is the Kronecker delta symbol.

To derive the explicit expression of the 3D strain field, Equation (1) is substituted into Equation (4). Neglecting higher-order terms that do not contribute to the total strain energy yields

(5) $$\begin{array}{cc} \varepsilon_{11} & ={\bar{\varepsilon }}_{11}+\xi y_{3}\kappa_{11}+\chi_{1,1}+\frac{1}{\xi }\chi_{1;1},\\ 2\varepsilon_{12} & =2{\bar{\varepsilon }}_{12}+\xi y_{3}\left(\kappa_{12}+\kappa_{21}\right)+\chi_{1,2}+\chi_{2,1}+\frac{1}{\xi }\left(\chi_{2;1}+\chi_{1;2}\right),\\ \varepsilon_{22} & ={\bar{\varepsilon }}_{22}+\xi y_{3}\kappa_{22}+\chi_{2,2}+\frac{1}{\xi }\chi_{2;2},\\ 2\varepsilon_{13} & =\chi_{1;3}+\chi_{3,1}+\frac{1}{\xi }\chi_{3;1},\\ 2\varepsilon_{23} & =\chi_{2;3}+\chi_{3,2}+\frac{1}{\xi }\chi_{3;2},\\ \varepsilon_{33} & =\chi_{3;3} \end{array}$$

where $\varepsilon_{ij}$ denotes the engineering strain components in the global Cartesian coordinate system, ${\bar{\varepsilon }}_{ij}$ the mid-plane (membrane) strain components, $\kappa_{ij}$ the curvature components, $\chi_{i}$ the warping functions, ${\left(\cdot \right)}_{,i}=\partial \left(\cdot \right)/\partial x_{i}$, ${\left(\cdot \right)}_{;i}=\partial \left(\cdot \right)/\partial y_{i}$, and

(6) $${\bar{\varepsilon }}_{\alpha \beta }\left(x_{1},x_{2}\right)=\frac{1}{2}\left({\bar{u}}_{\alpha ,\beta }+{\bar{u}}_{\beta ,\alpha }\right),\;\kappa_{\alpha \beta }\left(x_{1},x_{2}\right)=-{\bar{u}}_{3,\alpha \beta }$$

The strain energy of the SP-EHC can be expressed as follows:

(7) $$U=\int_{x_{1}}\int_{x_{2}}\frac{U_{\Omega }}{\Omega }dx_{1}dx_{2},$$

where $\frac{U_{\Omega }}{\Omega }$ denotes the strain energy density and Ω is the representative unit cell domain in the $x_{1}$-$x_{2}$ plane.

**Figure 2:**
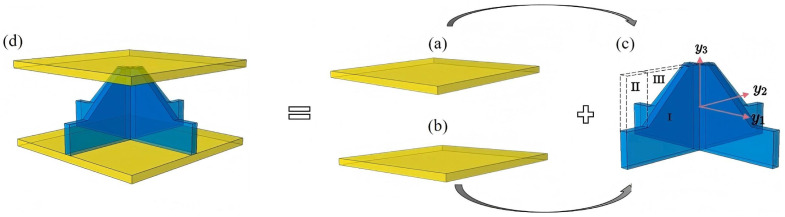
Schematic of VAM-based strain energy derivation for SP-EHC: (**a**) top facesheet, (**b**) bottom facesheet, (**c**) egg-crate core with local coordinate system and regions I–III, and (**d**) complete sandwich panel.

The explicit form of $U_{\Omega }$ is given by

(8) $$\begin{array}{cc} U_{\Omega }= & \int_{\frac{h_{2}}{2}}^{\frac{h_{2}}{2}+t_{f}}\int_{\frac{-L_{2}}{2}}^{\frac{L_{2}}{2}}\int_{\frac{-L_{1}}{2}}^{\frac{L_{1}}{2}}\sigma_{t}\varepsilon_{t}\;dy_{1}dy_{2}dy_{3}+\int_{-\frac{h_{2}}{2}-t_{f}}^{-\frac{h_{2}}{2}}\int_{\frac{-L_{2}}{2}}^{\frac{L_{2}}{2}}\int_{\frac{-L_{1}}{2}}^{\frac{L_{1}}{2}}\sigma_{b}\varepsilon_{b}\;dy_{1}dy_{2}\\ \; & +\;4\times (\int_{-\frac{h_{1}}{2}}^{\frac{h_{1}}{2}}\int_{-\frac{h_{1}}{2}}^{0}\int_{-\frac{t}{2}}^{\frac{t}{2}}\sigma_{I}\varepsilon_{I}dy_{1}dy_{2}dy_{3}-\int_{-\frac{h_{1}}{2}+h_{2}}^{\frac{h_{1}}{2}}\int_{-\frac{h_{1}}{2}}^{-\frac{h_{1}}{2}+b_{2}}\int_{-\frac{t}{2}}^{\frac{t}{2}}\sigma_{\mathrm{II}}\varepsilon_{\mathrm{II}}dy_{1}dy_{2}dy_{3}\\ \; & \;-\frac{1}{2}\int_{-\frac{h_{1}}{2}+h_{2}}^{\frac{h_{1}}{2}}\int_{-\frac{h_{1}}{2}+b_{2}}^{\frac{b_{1}}{2}}\int_{-\frac{t}{2}}^{\frac{t}{2}}\sigma_{\mathrm{III}\;}\varepsilon_{\mathrm{III}\;}dy_{1}dy_{2}dy_{3}) \end{array},$$

where the subscript *b* and *t* refer to the bottom and top face sheets, respectively; subscripts I, II, and III denote distinct regions within the core cell, as illustrated in Figure 2.

The virtual work $W_{3D}$ performed by external loads on the SP-EHC can be decomposed into two parts: the virtual work $W_{2D}$ related to the 2D-EPM, and the remaining part $W^{*}$, such that

(9) $$\begin{array}{cc} W_{3D} & =W_{2D}+W^{*}\\ \; & =\int_{s}\left(p_{i}{\bar{u}}_{i}+q_{\alpha }\delta u_{3,\alpha }\right)\;ds+\int_{s}\left(\langle f_{i}\chi_{i}\rangle +\tau_{i}\chi_{i}^{+}-\beta_{i}\chi_{i}^{-}\right)ds \end{array}$$

where $\chi_{i}^{-}$ and $\chi_{i}^{+}$ denote the warping displacements on the bottom and top surfaces, respectively; $p_{i}$ and $\tau_{i}$ represent the surface tractions on the bottom and top surfaces; $f_{i}$ is the body force. The distributed force contributions to the virtual work are expressed as follows:

(10) $$p_{i}=\left(f_{i}\right)+\tau_{i}+\beta_{i},\;q_{\alpha }=\frac{h}{2}\left(\beta_{\alpha }-\tau_{\alpha }\right)-\langle y_{3}f_{\alpha }\rangle$$

The original 3D kinetic energy of SP-EHC can be expressed as follows:

(11) $$K_{3D}=K_{2D}+K^{*}$$

where

(12) $$K_{2D}=\frac{1}{2}\int_{s}\left(\rho {\bar{V}}^{T}\bar{V}+2\alpha^{T}\rho \xi \bar{V}+\alpha^{T}\Phi \alpha \right)ds$$

(13) $$K^{*}=\frac{1}{2}\int_{\nu }\rho \left[{\left(\dot{\chi }+\dot{\chi }\right)}^{T}\left(\alpha \xi +\dot{\chi }\right)+2{\left(V+\alpha \xi \right)}^{T}\left(\alpha \chi +\dot{\chi }\right)\right]dv$$

where *V* and α denote the absolute velocity and angular velocity, respectively, and *v* is the volume occupied by the sandwich panel. The corresponding quantities are defined as follows:

(14) $$\dot{x}=\partial x/\partial t,\;\xi ={\left[0\;0\;x_{3}\right]}^{T},\;\rho \xi ={\left[0\;0\;\langle x_{3}\rho \rangle \right]}^{T},\;\Phi =\left[\begin{array}{ccc} 0 & 0 & 0\\ 0 & 0 & 0\\ 0 & 0 & \langle x_{3}^{2}\rho \rangle \end{array}\right]$$

The parameters ρ and $\bar{\rho }$ represent the mass densities of 3D-FEM and 2D-EPM, respectively. The equivalent mass density $\bar{\rho }$ can be determined by assuming ideal face sheet–core bonding, while neglecting the mass contribution of internal walls within the cellular structure, such as

(15) $$\bar{\rho }=\frac{m_{c}+m_{f}}{h\cdot l_{1}^{2}}=\frac{4\rho_{c}V_{c}+2\rho_{f}V_{f}}{\left(h_{1}+2t_{f}\right)\cdot l_{1}^{2}}$$

where $\rho_{c}$ and $\rho_{f}$ are the mass densities of the core and face sheets, respectively; $V_{f}=l_{1}^{2}\cdot t_{f}$ represents the volume of face sheet. Based on geometric modeling, the core volume $V_{c}$ can be expressed as follows:

(16) $$V_{c}=\left[\frac{l_{1}}{2}\times h_{1}-b_{2}\left(h_{1}-h_{2}\right)-\frac{1}{2}\left(\frac{l_{1}}{2}-b_{2}-\frac{b_{1}}{2}\right)\left(h_{1}-h_{2}\right)\right]\cdot t$$

The elastic dynamic behavior of SP-EHC satisfies Hamilton’s principle, which can be stated as follows:

(17) $$\int_{t_{1}}^{t_{2}}\left[\delta \left(K_{2D}+K^{*}-U\right)+\delta W_{2D}+\delta W^{*}\right]dt=0$$

The total strain energy of a periodic unit cell subjected to a macroscopic strain field $\varepsilon_{ij}$ is given by

(18) $$\Pi \left[\chi_{i}\right]=\frac{1}{2}\int_{\Omega }C_{ijkl}\left(\varepsilon_{ij}-\chi_{i;j}\right)\left(\varepsilon_{kl}-\chi_{k;l}\right)d\Omega$$

where $C_{ijkl}$ is the elasticity tensor of the constituent material.

The governing equation for the variation in $\chi_{i}$ can be expressed as follows:

(19) $$\min_{\chi_{i}\in Eq.\left(3\right)}\langle \varepsilon^{T}D\varepsilon \rangle$$

where ε denotes the strain vector and D can be expressed as [44]

(20) $$D=\frac{1}{\Omega }\int_{\Omega }B^{T}CBd\Omega$$

where C is the elasticity tensor of the constituent material and B is the transformation matrix relating global strain to local warping-induced strain fields.

Directly solving the minimization problem in Equation (19) poses significant challenges, particularly in the context of fully 3D elastic analyses. However, by conducting an asymptotic analysis of the transformed expressions within the equivalent formulation, a potential solution for $\chi_{i}$ can be derived without imposing restrictive assumptions on the displacement field or material behavior. The detailed procedure for this asymptotic approach will be presented in the following section.

### 2.2. Equivalent Analysis Based on the VAM

To accurately reduce the original 3D problem to a 2D equivalent model, it is essential to recover the strain energy stored in the 3D structure within the 2D formulation. This is most effectively achieved by leveraging small geometric or material parameters inherent to the structure and applying a stepwise transformation via asymptotic analysis. Within this framework, the orders of magnitude of all quantities involved in the expansion can be expressed as follows:

(21) $$\varepsilon_{\alpha \beta }∼h\kappa_{\alpha \beta }∼\eta ,\;f_{3}∼\mu {\left(\frac{h}{L_{1}}\right)}^{2}\eta ,\;f_{\alpha }∼\mu \left(\frac{h}{L_{1}}\right)\eta ,\;m_{\alpha }∼\mu h\left(\frac{h}{L_{1}}\right)\eta$$

where μ and *h* represent the characteristic material property scale and geometric scale, respectively; η is a small perturbation parameter.

By analyzing the scaling orders in Equation (21), it becomes evident that the applied loads contribute at higher orders. As a result, terms corresponding to zero-order energy equivalence can be reasonably neglected. Referring to Equation (5), the strain field under the zero-order approximation can be expressed in matrix form as follows:

(22) $$\varepsilon =\Gamma_{\chi }\chi +\Gamma_{\varepsilon }\varepsilon$$

where $\chi ={\left[\begin{array}{ccc} \chi_{1} & \chi_{2} & \chi_{3} \end{array}\right]}^{T},\;\varepsilon ={\left[\begin{array}{cccccccc} \varepsilon_{11} & 2\varepsilon_{12} & \varepsilon_{22} & \kappa_{11} & 2\kappa_{12} & \kappa_{22} & 2\gamma_{13} & 2\gamma_{23} \end{array}\right]}^{T}$, and

(23) $$\Gamma_{\chi }=\left[\begin{array}{ccc} \frac{\partial }{\partial y_{1}} & 0 & 0\\ \frac{\partial }{\partial y_{2}} & \frac{\partial }{\partial y_{1}} & 0\\ 0 & \frac{\partial }{\partial y_{2}} & 0\\ \frac{\partial }{\partial x_{3}} & 0 & \frac{\partial }{\partial y_{1}}\\ 0 & \frac{\partial }{\partial x_{3}} & \frac{\partial }{\partial y_{2}}\\ 0 & 0 & \frac{\partial }{\partial x_{3}} \end{array}\right],\;\Gamma_{\varepsilon }=\left[\begin{array}{ccccccccc} 1 & 0 & 0 & \xi y_{3} & 0 & 0 & 0 & 0 & 0\\ 0 & 1 & 0 & 0 & \xi y_{3} & 0 & 0 & 0 & 0\\ 0 & 0 & 1 & 0 & 0 & \xi y_{3} & 0 & 0 & 0\\ 0 & 0 & 0 & 0 & 0 & 0 & 1 & 0 & 0\\ 0 & 0 & 0 & 0 & 0 & 0 & 0 & 1 & 0\\ 0 & 0 & 0 & 0 & 0 & 0 & 0 & 0 & 1 \end{array}\right]$$

To obtain a more general approximation, the warping functions $\chi_{i}\left(x_{1},x_{2};y_{1},y_{2},y_{3}\right)$ are discretized using a numerical method (such as finite element interpolation) as

(24) $$\chi_{i}\left(x_{1},x_{2};y_{1},y_{2},y_{3}\right)=S\left(y_{1},y_{2},y_{3}\right)N\left(x_{1},x_{2}\right)$$

where *S* is the shape function matrix depending on microscopic coordinates; *N* is the shape function matrix formed by the nodal values of $\chi_{i}$.

By substituting Equations (24) into (9) and (22), the weak form of the zero-order approximate energy can be expressed as follows:

(25) $$\Pi_{\Omega }=\frac{1}{2\Omega }\left(N^{T}D_{h\chi }N+2N^{T}D_{h\varepsilon }\varepsilon +\varepsilon^{T}D_{\varepsilon }\varepsilon \right)$$

where

(26) $$\begin{array}{cc} D_{h\chi } & =\int_{\Omega }{\left(\Gamma_{h}S\right)}^{T}D\left(\Gamma_{h}S\right)\;d\Omega ,\\ D_{h\varepsilon } & =\int_{\Omega }{\left(\Gamma_{h}S\right)}^{T}D\Gamma_{\varepsilon }\;d\Omega ,\\ D_{\varepsilon } & =\int_{\Omega }\Gamma_{\varepsilon }^{T}D\Gamma_{\varepsilon }\;d\Omega . \end{array}$$

By applying the averaging constraint condition in Equation (3) and minimizing Equation (25), a linear system is obtained as follows:

(27) $$D_{h\chi }N=-D_{h\varepsilon }\varepsilon$$

Since *N* depends linearly on the 2D plate strain ε, and this relationship is independent of the strain magnitude, the solution for the warping parameters can be symbolically expressed as follows:

(28) $$N=N_{0}\varepsilon$$

Substituting Equation (28) into Equation (25) yields the final form of the strain energy density stored within the unit cell:

(29) $$\Pi_{\Omega }=\frac{1}{2\Omega }\varepsilon^{T}\left(N_{0}^{T}D_{h\varepsilon }+D_{\varepsilon }\right)\varepsilon =\frac{1}{2}\varepsilon^{T}D_{E}\varepsilon$$

where $D_{E}$ denotes the effective stiffness matrix, characterizing the equivalent elastic properties of the unit cell that incorporates complex microstructural materials and geometry.

### 2.3. Transformation into the Reissner–Mindlin Model

Although the zero-order asymptotic strain energy correction in Equation (29) is mathematically valid, an additional step is required to map the current approximate energy expression to the classical Reissner–Mindlin plate model. The generalized form of the Reissner–Mindlin strain energy is given by

(30) $$2\Pi_{R}=R^{T}XR+\gamma^{T}G\gamma +2R^{T}F_{R}$$

In Equation (30), the Reissner–Mindlin model is obtained by eliminating the coupling terms between the membrane–bending strain vector $\varepsilon ={\left[\varepsilon_{11},2\varepsilon_{12},\varepsilon_{22},\kappa_{11},\kappa_{12},\kappa_{21},\kappa_{22}\right]}^{T}$ and the transverse shear strain vector $\gamma ={\left[2\gamma_{13},2\gamma_{23}\right]}^{T}$. The full strain energy expression can be rewritten as follows:

(31) $$\begin{array}{c} 2\Pi_{a}=\varepsilon^{T}\bar{D}\varepsilon =\varepsilon^{T}\bar{A}\varepsilon +2{\bar{\varepsilon }}^{T}\bar{B}\gamma +\gamma^{T}\bar{C}\gamma \\ ={\bar{\varepsilon }}^{T}\left(\bar{A}-{\bar{BC}}^{T}{\bar{B}}^{T}\right)\bar{\varepsilon }+{\left(\gamma +{\bar{C}}^{-1}{\bar{B}}^{T}\bar{\varepsilon }\right)}^{T}\dot{C}\left(\gamma +{\bar{C}}^{-1}{\bar{B}}^{2}\right) \end{array}$$

In addition, the strain energy expression must include the work conjugate to the external load term *F*, which is expressed as follows:

(32) $$F=-N_{0}^{T}\tau -N_{0}^{T}\beta -\langle N_{0}^{T}f\rangle$$

The total energy of the 2D-EPM can be expressed using the variable transformations $R=\bar{\varepsilon }$ and $\bar{\gamma }=\gamma +C^{-1}B^{T}\bar{\varepsilon }$, leading to

(33) $$2\Pi_{R}=R^{T}XR+{\bar{\gamma }}^{T}G\bar{\gamma }+2R^{T}F_{R}$$

where *G* is shear stiffness, and

(34) $$X=A-BC^{-1}B^{T},\;G=C,\;F_{R}=F$$

Due to the axial and planar symmetry of the SP-EHC, certain stiffness components are eliminated, leading to the final constitutive relation of the 2D-EPM:

(35) $$\left[\begin{array}{c} N_{11}\\ N_{22}\\ N_{12}\\ M_{11}\\ M_{22}\\ M_{12}\\ Q_{1}\\ Q_{2} \end{array}\right]=\left[\begin{array}{cccccccc} A_{11} & A_{12} & 0 & B_{11} & B_{12} & 0 & 0 & 0\\ \; & A_{22} & 0 & B_{12} & B_{22} & 0 & 0 & 0\\ \; & \; & A_{66} & 0 & 0 & B_{66} & 0 & 0\\ \; & \; & & D_{11} & D_{12} & 0 & 0 & 0\\ \; & \;\mathrm{sym}\; & \; & & D_{22} & 0 & 0 & 0\\ \; & \; & & \; & & D_{66} & 0 & 0\\ \; & \; & & \; & & \; & C_{11} & 0\\ \; & \; & & \; & & \; & & C_{22} \end{array}\right]\left[\begin{array}{c} \varepsilon_{11}\\ \varepsilon_{22}\\ 2\varepsilon_{12}\\ \kappa_{11}\\ \kappa_{22}\\ 2\kappa_{12}\\ 2\gamma_{13}\\ 2\gamma_{23} \end{array}\right]$$

where $N_{\alpha \beta }$, $M_{\alpha \beta }$, and $Q_{\alpha }$ (α,β=1,2) denote the in-plane forces, bending moments, and shear force, respectively.

The principal advantage of this method lies in its ability to construct the Reissner–Mindlin plate model directly from the zero-order asymptotic approximation, thereby eliminating the need for higher-order expansions or external transformation procedures. This approach enables the macroscopic behavior to be explicitly described through variable expressions derived from the asymptotic system, which inherently capture all 2D field variables in the in-plane coordinates $x_{1}$ and $x_{2}$. Consequently, the overall problem formulation is greatly simplified while preserving the essential mechanical characteristics of the original 3D structure.

### 2.4. Local Field Recovery

In addition to capturing macroscopic responses, evaluating the local field behavior within the original microstructure is often essential. Based on the results of the macroscopic structural analysis, the local displacement field can be reconstructed as

(36) $$u_{i}={\bar{u}}_{i}+\left[\begin{array}{ccc} {\bar{u}}_{1,1} & {\bar{u}}_{1,2} & {\bar{u}}_{1,3}\\ {\bar{u}}_{2,1} & {\bar{u}}_{2,2} & {\bar{u}}_{2,3}\\ {\bar{u}}_{3,1} & {\bar{u}}_{3,2} & {\bar{u}}_{3,3} \end{array}\right]\left[\begin{array}{c} y_{1}\\ y_{2}\\ y_{3} \end{array}\right]+\xi_{i}$$

where $u_{i}$ and ${\bar{u}}_{i}$ denote the displacements of 3D and 2D models, respectively; $y_{i}$ denote the local coordinates within the unit cell; and $\xi_{i}$ is the warping function.

The local strain field can be reconstructed as follows:

(37) $$\begin{array}{cc} \; & \varepsilon_{e}={\left[\varepsilon_{11}\;\varepsilon_{22}\;2\varepsilon_{12}\right]}^{T}=\varepsilon +\xi y_{3}\kappa \\ \; & 2\xi_{s}={\left[2\varepsilon_{13}\;2\varepsilon_{23}\right]}^{T}=-\left(\varepsilon +\xi y_{3}\kappa \right){\bar{C}}_{es}^{*}C_{s}^{-1}\\ \; & \varepsilon_{t}=\varepsilon_{33}=-\left(\varepsilon +\xi y_{3}\kappa \right){\bar{C}}_{et}^{*}{\bar{C}}_{t}^{-1} \end{array}$$

where $\varepsilon_{ij}$ denotes the strain components of the 3D model.

According to the 3D constitutive relationship (Hooke’s law), the local stress field within the unit cell can be obtained as follows:

(38) $$\sigma =D_{E}\varepsilon$$

**Figure 3:**
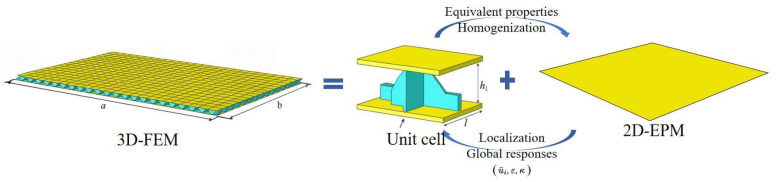
Schematic of 2D-EPM for SP-EHC analysis with unit-cell homogenization and localization.

In summary, the local stress and strain fields within the unit cell can be accurately reconstructed using the generalized macroscopic strain ε, curvature κ, and displacement ${\bar{u}}_{i}$ within the equivalent model framework, as shown in Figure 3. This multi-scale reconstruction approach ensures that detailed micro-scale mechanical responses are captured while leveraging the computational efficiency of the reduced two-dimensional formulation.

## 3. Dynamic Verification

In practical engineering applications, sandwich panels are often subjected to stochastic dynamic loads, including wind pressure, seismic excitation, and vehicular vibrations, which can critically affect their dynamic behavior, buckling resistance, and fatigue life. Consequently, an accurate assessment of their vibrational response across different modes is essential to ensure structural safety and reliability. However, full three-dimensional (3D) modeling of the SP-EHC entails significant computational effort due to the complexity of its geometry and material composition, thereby limiting analysis efficiency and model scalability. To address these challenges, a VAM-based equivalent model was employed to predict the free-boundary vibration behavior of the SP-EHC.

### 3.1. Material Property Testing

**Figure 4:**
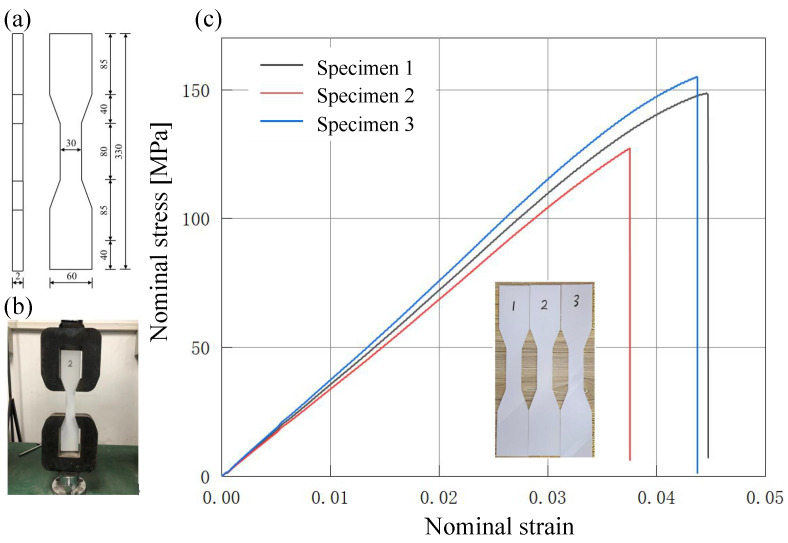
(**a**) Dimensions of the dog-bone-shaped tensile specimen (mm), (**b**) experimental setup for uni-axial tensile testing, (**c**) nominal stress–strain curves of PLA specimens under uni-axial tension.

The mechanical properties of the 3D-printed PLA material were determined through uni-axial tensile tests conducted in accordance with the GB/T 1040.2-2006 standard using a universal testing machine. Dog-bone-shaped specimens, with dimensions shown in Figure 4a, were carefully aligned and mounted to ensure vertical positioning and prevent slippage. A small preload was applied to eliminate potential seating effects. The tests were performed at a constant displacement rate of 5 mm/min until failure. Load and displacement were recorded in real time, while strain was measured using an extensometer to ensure high accuracy. Nominal stress–strain curves were obtained from the load–displacement data, as illustrated in Figure 4c. The linear elastic region was identified by excluding initial seating and post-yield nonlinearity, and the elastic modulus was calculated as the slope of a least-squares linear fit over this range. The average elastic modulus, determined from three specimens, was 2413 MPa. The Poisson’s ratio and density were measured separately, yielding values of 0.39 and 1.30 g/cm^3^, respectively.

### 3.2. Free-Boundary Vibration Analysis

**Figure 5:**
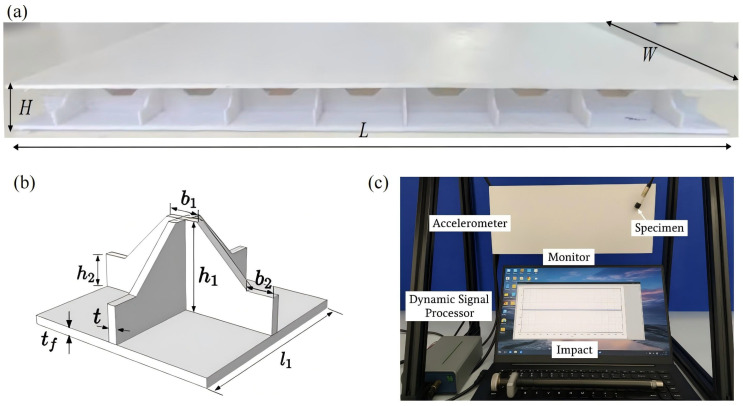
(**a**) Photograph of the fabricated 3D egg-crate core sandwich panel; (**b**) schematic of a unit cell with geometric parameters; (**c**) experimental setup for free-boundary vibration testing.

In modal testing, boundary constraints are typically applied using mechanical fixtures; however, the elastic deformation of these fixtures can introduce additional compliance, adversely affecting the accuracy of measured modal frequencies and mode shapes. This deviation from ideal boundary conditions complicates high-precision modal analysis. To mitigate such boundary interference and ensure reliable validation of the VAM-based 2D-EPM, all-PLA specimens were fabricated using 3D printing technology. Modal vibration tests were conducted under free boundary conditions to eliminate fixture-induced artifacts. The specimens consisted of eight and four unit cells in the $x_{1}$ and $x_{2}$ directions, respectively, with unit cell dimensions listed in Table 1. The overall dimensions of the specimen were *L* = 360 mm, *W* = 180 mm, and *H* = 24 mm, as illustrated in Figure 5a. This dense measurement configuration significantly improves the spatial resolution of modal data, thereby enhancing the accuracy of numerical model validation.

The free-boundary vibration test setup for the SP-EHC specimen is shown in Figure 5c. To simulate free boundary conditions, the specimen was suspended from a gantry frame using two nylon cords. An accelerometer was mounted at a fixed location on the specimen surface to capture real-time response signals. The structure was excited using an impact hammer, and both force and acceleration time histories were recorded via a dynamic signal analyzer and transmitted to a computer for processing. A fast Fourier transform (FFT) was then applied to convert the time-domain data into the frequency domain, allowing for extraction of the natural frequencies. In the numerical simulation, a frequency analysis step within the linear perturbation module was used to compute the first seven eigenfrequencies. The model was assigned completely free boundary conditions, and the interaction between the honeycomb core and face sheets was represented using “Tie” constraints. The three-dimensional model comprised 84,125 C3D10 elements, while the two-dimensional model employed 1525 S4R elements.

**Figure 6:**
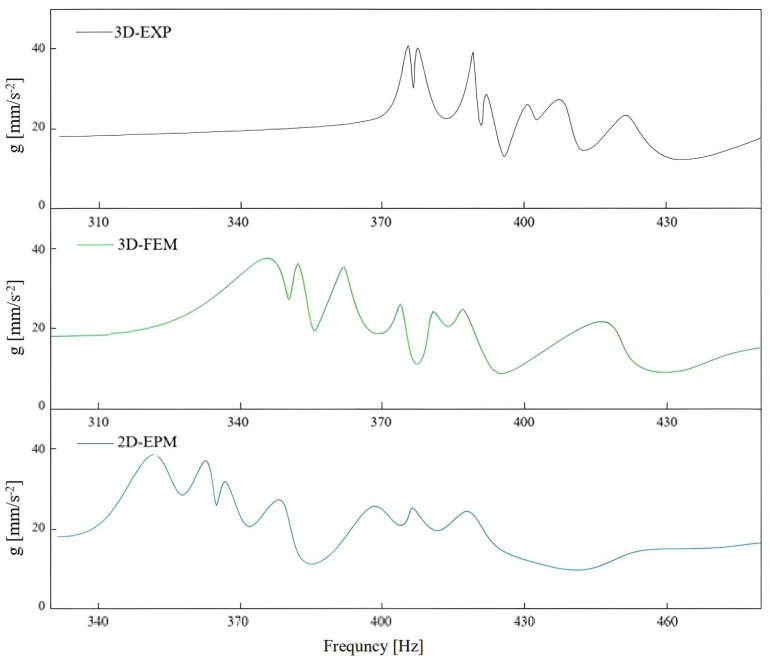
Comparison of frequency-domain responses of SP-EHC obtained from 3D-EXP, 3D-FEM, and 2D-EPM.

Figure 6 shows seven distinct energy peaks, corresponding to the first through seventh modal frequencies of the SP-EHC under free boundary conditions, within the 310–430 Hz range. Each modal frequency was accurately identified using the half-power bandwidth method. The prominent peaks reflect resonance responses at specific frequencies, highlighting the structure’s characteristic modal behavior.

**Figure 7:**
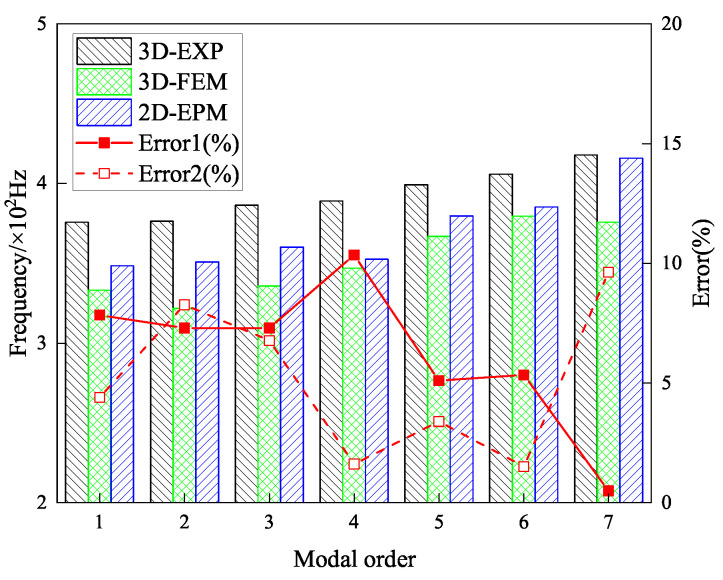
Comparison of natural frequencies and modal errors for the first seven modes obtained from 3D-EXP, 3D-FEM, and 2D-EPM. Errors 1 and 2 refer to the deviations in the 3D-FEM and 2D-EPM results from the 3D-EXP data, respectively.

Figure 7 shows the first seven elastic mode frequencies, increasing with mode order. The seventh mode occurs at 420.27 Hz, about 1.12 times the first mode’s frequency of 375.73 Hz. Comparing frequencies from the three methods, the experimental results are the highest, followed by the 2D-EPM, with the 3D-FEM predicting the lowest. The higher experimental frequencies are due to the added stiffness of the nylon cord suspension, while the lower 3D-FEM frequencies result from localized face sheet vibrations at lower modes. In contrast, the 2D-EPM provides a balanced prediction. Specifically, the 2D-EPM prediction of the first mode frequency (356.94 Hz) differs by only 5% from the EXP result (375.73 Hz) and 2.5% from the 3D-FEM (348.01 Hz).

Despite good agreement between the 2D-EPM, 3D-FEM, and experimental results, minor discrepancies in frequency-domain responses were noted, particularly between 3D-FEM and EXP data. These differences stem from factors such as the nylon cord suspension in the experiment, which introduces additional stiffness and damping; anisotropic properties and imperfections in the PLA material; accelerometer mass effects; and slight variations in physical constraints and bonding in the 3D-FEM. Nevertheless, as shown in Figure 7, the maximum deviation in modal frequencies is 10.23%, with all other modes showing errors below 10%, which are acceptable for engineering applications. These results validate the VAM-based 2D-EPM’s accuracy and computational efficiency in predicting the dynamic behavior of the SP-EHC.

**Figure 8:**
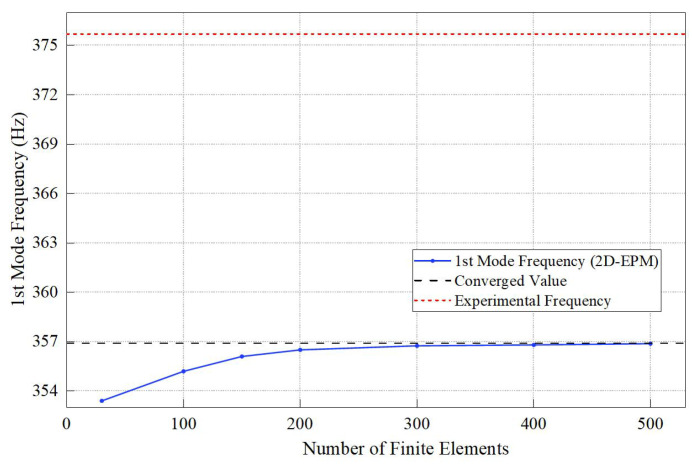
Mesh convergence study for the 2D-EPM: 1st mode frequency as a function of finite element number. The result stabilizes for meshes with more than 200 elements, confirming model consistency.

To evaluate the numerical consistency and robustness of the proposed equivalent model, a mesh convergence study was conducted for the 2D-EPM. The first mode frequency was computed for finite element numbers ranging from 50 to 500. As shown in Figure 8, the frequency stabilizes with mesh refinement and, for more than 200 elements, the variation is under 1.5%, indicating convergence. The final converged value (356.94 Hz) deviates by less than 5% from the experimental result (375.73 Hz), confirming the model’s validity and efficiency. This demonstrates the 2D-EPM’s high fidelity and stability across discretization levels, suitable for engineering applications.

## 4. Static Verification

Static analysis plays a critical role in evaluating the structural integrity and load-bearing capacity of sandwich panels under various boundary and loading conditions. In this chapter, the static performance of the SP-EHC is examined using both the 3D-FEM and the VAM-based 2D-EPM. The investigation covers buckling behavior under in-plane compression, out-of-plane bending with and without initial geometric imperfections, and localized stress and displacement field reconstruction. In the experimental setup, the face sheets were fabricated using PLA material due to ease of 3D printing and boundary control. For static verification, CFRP face sheets were used to represent real-world applications. This material distinction has no impact on the validity of the proposed model.

### 4.1. Structural Modeling

**Figure 9:**
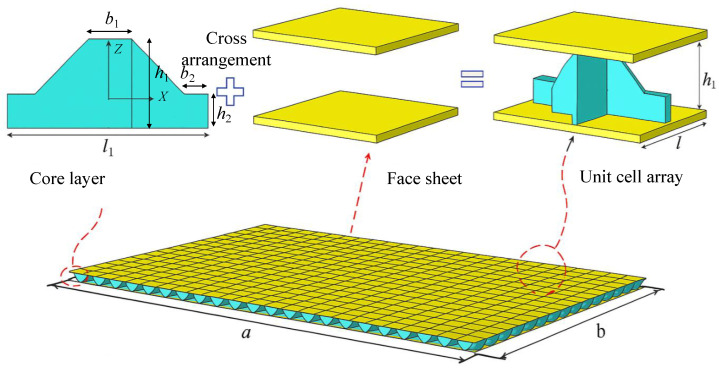
Schematic illustration of the SP-EHC construction. The panel consists of two CFRP face sheets and an aluminum core layer formed by a periodic array of unit cells. Each unit cell features a cross-shaped configuration composed of orthogonally arranged structural elements.

**Figure 10:**
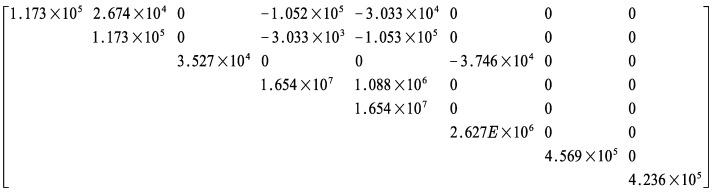
Transformation of the theoretical stiffness matrix into its numerical representation (unit: SI).

The structural parameters used for finite element validation are provided in Table 1. Figure 9 illustrates that the 3D-FEM consists of a metallic core layer sandwiched between upper and lower carbon-fiber-reinforced polymer (CFRP) face sheets. The unit cell is periodically replicated 21 times along the $x_{1}$ direction and 16 times along the $x_{2}$ direction, forming a full-scale SP-EHC. Each face sheet comprises eight CFRP plies with a thickness of 0.25 mm per ply and a symmetric layup sequence of ${\left[0/90/90/0\right]}_{s}$, designed to enhance stiffness and load-bearing capacity in both principal directions. The matrix material is epoxy resin with a fiber volume fraction of 60%. The core layer is fabricated from aluminum, and its material properties are summarized in Table 2. The equivalent stiffness matrix, derived using the VAM, serves as the primary input for constructing the 2D-EPM, as illustrated in Figure 10. It includes the in-plane stiffness (A), bending stiffness (D), bending–membrane coupling stiffness (B), and shear stiffness (C), as shown in Figure 10. These submatrices form a block matrix used in the constitutive relation (Equation (35)), linking generalized stress resultants to strain fields. This matrix is then implemented in the finite element simulation of the 2D-EPM to calculate the global response under static loading.

It is worth noting that the equivalent in-plane shear modulus (*G*) obtained via the VAM-based model effectively represents the shear stiffness of the egg-crate honeycomb core. Based on classical sandwich beam theory, the panel cross-section can be idealized as an I-beam, where the core (web) carries over 80% of the shear force while the face sheets (flanges) mainly resist bending. Hence, *G* can be approximated as the in-plane shear modulus of the core. This modeling assumption is standard in sandwich structure analysis and supports the validity of the proposed 2D equivalent model in capturing core-dominated shear behavior.

### 4.2. Buckling Analysis Under In-Plane Loading

Buckling performance analysis of composite sandwich panels is of considerable engineering significance. Owing to their high strength-to-weight ratio, these structures are extensively employed in aerospace, civil engineering, and transportation, offering superior load-bearing capacity and structural stability. Buckling analysis facilitates the evaluation of instability risks under diverse loading and boundary conditions, providing a theoretical basis for structural optimization and safety evaluation.

**Figure 11:**
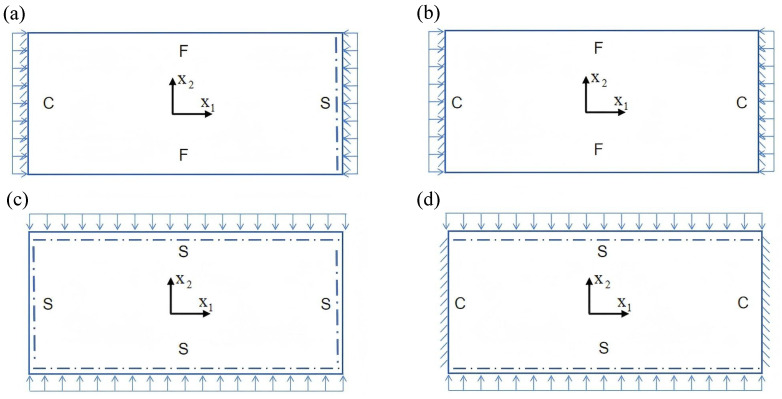
Boundary condition configurations for rectangular composite sandwich panels under in-plane loading: (**a**) Case 1: CSFF, (**b**) Case 2: CCFF, (**c**) Case 3: SSSS, and (**d**) Case 4: CCSS.

To evaluate the applicability of the 2D-EPM for buckling prediction, analyses were performed using both 3D-FEM and 2D-EPM under four representative boundary conditions, as shown in Figure 11. In the figure, C, S, and F represent clamped, simply supported, and free edges, respectively. In Cases 1 (CSFF) and 2 (CCFF), compressive loads were applied along the short edge, whereas in Cases 3 (SSSS) and 4 (CCSS), loading was applied along the long edge. Symmetric compression along the long edge is commonly employed to investigate the buckling behavior of sandwich panels under longitudinal loading. In practical applications, these panels often experience bi-axial compression in the longitudinal direction due to self-weight, wind loads, and other external forces, potentially leading to in-plane buckling and a significant reduction in load-carrying capacity. Simulations under this loading condition enable effective evaluation of critical buckling loads, deformation characteristics, and failure modes, offering valuable insights for structural design.

Conversely, symmetric compression along the short edge examines the panel’s response to transverse loading. Scenarios such as lateral wind pressure or accidental impacts can induce local crushing or buckling near the short edges. This analysis facilitates a detailed assessment of local stiffness, compressive strength, and stability, thereby supporting safety evaluations under extreme loading conditions. A total compressive load of 1 N was applied at the boundaries in all cases; hence, the buckling eigenvalues obtained from the finite element analysis directly correspond to the critical buckling loads.

Table 3 compares the predicted first-order critical buckling loads and corresponding buckling modes of the SP-EHC under four typical boundary conditions, obtained using the 2D-EPM and 3D-FEM. The results indicate that the critical buckling loads predicted by the two models show minimal discrepancies, with a maximum deviation of no more than 6%. Moreover, the buckling modes exhibit high morphological agreement, demonstrating that the 2D-EPM offers reliable accuracy and applicability for predicting the first-order buckling behavior of sandwich panels.

Detailed comparisons reveal that the critical buckling load in Case 2 is approximately twice that of Case 1, indicating that enhanced boundary constraints significantly improve structural stability. In addition, the buckling mode shapes reveal that stress concentration regions tend to shift toward the less constrained edges, indicating a higher likelihood of local instability initiating in areas with weaker restraint. A similar trend is observed between Cases 3 and 4, further underscoring the critical role of boundary conditions in influencing buckling behavior.

Notably, in the comparison between Cases 2 and 3, Case 3 exhibits a higher critical buckling load despite having weaker boundary constraints. This seemingly counterintuitive result is primarily attributed to differences in loading direction. When compression is applied along the long edge, the panel benefits from greater bending stiffness and improved buckling resistance. Additionally, the increased structural flexibility in this direction promotes stress redistribution, thereby reducing the likelihood of local buckling. In contrast, although Case 2 features stronger constraints under short-edge compression, it is more prone to stress concentrations and localized deformations, increasing its susceptibility to instability. These findings indicate that critical buckling performance is governed not only by boundary conditions but also by loading direction and structural geometry. Consequently, enhancing boundary support in conjunction with optimizing the loading path offers an effective strategy for improving the load-bearing capacity and buckling stability of sandwich panels.

**Figure 12:**
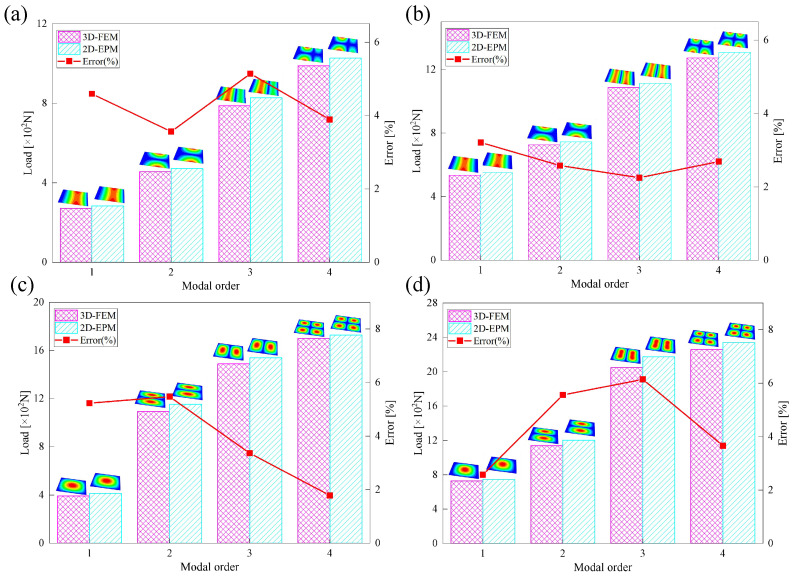
Comparison of the first four buckling loads predicted by 2D-EPM and 3D-FEM under (**a**) Case 1: CSFF, (**b**) Case 2: CCFF, (**c**) Case 3: SSSS, and (**d**) Case 4: CCSS.

Considering only the first-order buckling load may not fully capture the structural stability under realistic loading scenarios. Higher-order buckling modes often emerge at increased load levels and can significantly affect the overall instability mechanism, particularly under specific boundary or geometric conditions. Figure 12 shows that the first four buckling mode shapes predicted by the 2D-EPM and 3D-FEM demonstrate strong consistency across various boundary conditions. The relative errors in the corresponding buckling loads remain within 10%, confirming the accuracy and effectiveness of the 2D-EPM in capturing higher-order buckling behavior.

The buckling response of the SP-EHC is highly sensitive to both boundary conditions and loading direction. The number and arrangement of clamped edges significantly influence the critical buckling load and mode shapes: increasing the number of fixed boundaries enhances the overall structural stiffness, elevates the critical load, and tends to localize failure near the constrained regions. In contrast, a higher proportion of free edges leads to more complex buckling deformations and a more dispersed failure pattern. The loading direction also plays a critical role. Under long-edge compression with free boundaries, buckling modes are more evenly distributed across the panel. In comparison, short-edge compression induces instability localized near the free edges, resulting in more intricate and concentrated deformation patterns.

### 4.3. Out-of-Plane Bending Analysis Without Initial Displacement

**Figure 13:**
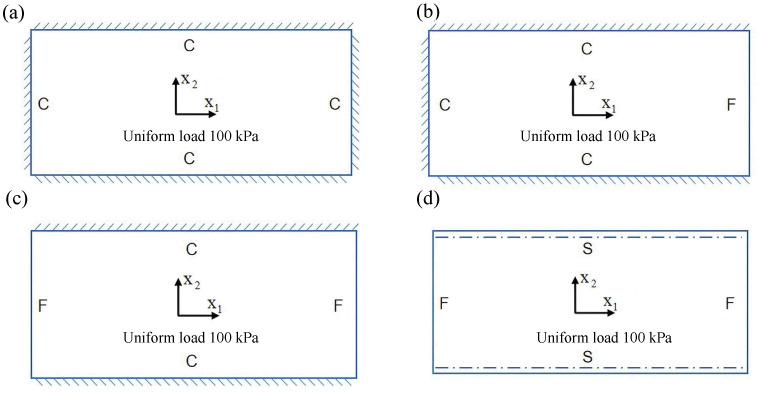
Four typical boundary conditions for out-of-plane bending analysis of SP-EHC under a uniform load of 100 kPa: (**a**) Case 5: CCCC, (**b**) Case 6: CCCF, (**c**) Case 7: CCFF, and (**d**) Case 8: SSFF.

Figure 13 illustrates four typical boundary conditions used to evaluate the out-of-plane bending deformation of the SP-EHC under a uniformly distributed load. Cases 5 to 8 correspond to CCCC, CCCF, CCFF, and SSFF boundary conditions, all subjected to a uniform pressure of 100 kPa with zero initial displacement. A general static analysis step was performed using the ABAQUS software to valuate the maximum displacement and overall deformation behavior of the sandwich panels. Table 4 presents the resulting displacement contours and the relative errors in maximum displacement for each case.

The analysis results reveal that maximum displacement increases progressively as boundary constraints are relaxed—from CCCC to SSFF BCs—indicating that the CCCC BCs provide the greatest out-of-plane bending stiffness while SSFF BCs offer the least. Specifically, the maximum displacement under SSFF BCs is nearly five times greater than that under CCCC BCs. This is attributed to the lack of moment restraint in the SSFF BCs, where simply supported edges allow for free rotation and bending, resulting in significantly larger deformations. The comparison of displacement contours shows strong agreement between the 3D-FEM and 2D-EPM in both deformation patterns and numerical values. The relative error in maximum displacement across all boundary conditions ranges from 2.05% to 4.15%, remaining within acceptable engineering limits. These results confirm that the 2D-EPM provides accurate and reliable predictions of the out-of-plane bending behavior of the SP-EHC under various boundary conditions.

**Figure 14:**
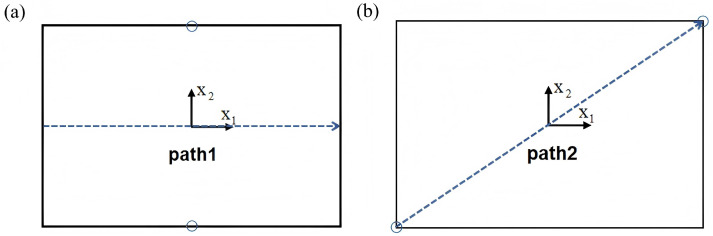
Definition of typical displacement extraction paths for deformation analysis. (**a**) Path 1 is aligned along the longitudinal midline of the sandwich panel, (**b**) Path 2 is set along the diagonal of the panel.

To systematically evaluate the accuracy of the 2D-EPM in predicting the out-of-plane mechanical performance of the SP-EHC, representative displacement extraction paths were designed in addition to the maximum displacement analysis. As illustrated in Figure 14, two characteristic paths were selected for detailed evaluation. Path 1 follows the midline along the longitudinal axis of the sandwich panel, capturing deformation along the primary load-bearing direction. Path 2 extends diagonally across the panel, providing a comprehensive view of global deformation patterns under complex loading conditions. These two paths enable systematic extraction of displacement–path distribution curves under four representative boundary conditions.

Displacement distribution curves were extracted from Abaqus/CAE using a path-based post-processing method. A path was defined along the mid-span or diagonal-line of the sandwich panel, and displacement values were sampled using the XY Data from Path tool. The data were exported in CSV format and plotted with Origin software. This method allows for clear visualization of deformation trends and local displacement gradients, ensuring consistent comparison between 2D-EPM and 3D-FEM results.

**Figure 15:**
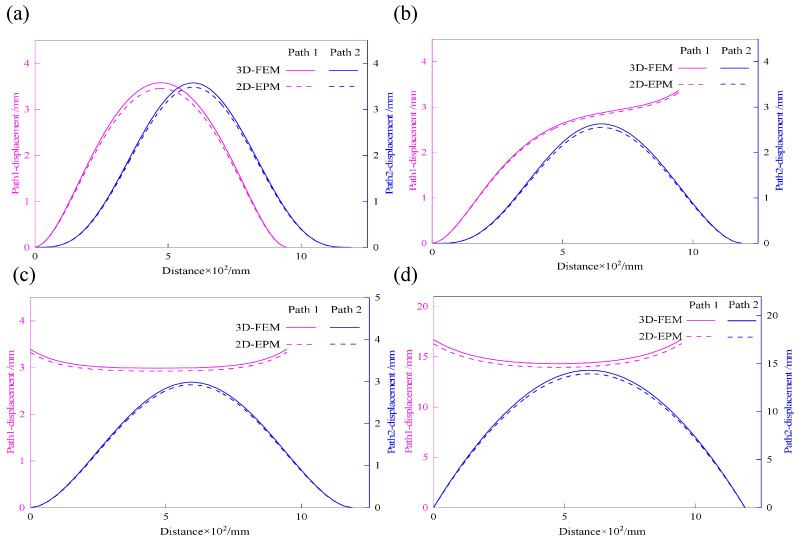
Comparison of displacement–path curves of 3D-FEM and 2D-EPM under different cases: (**a**) Case 5, (**b**) Case 6, (**c**) Case 7, and (**d**) Case 8.

Figure 15 shows the displacement distribution curves along Path 1 and Path 2 under four representative boundary conditions, comparing results from the 3D-FEM and 2D-EPM. Analysis of the curves reveals that, for Cases 5 (CCCC), 7 (CCFF), and 8 (SSFF), the displacement profiles along Path 1 exhibit pronounced symmetry in the $x_{2}$ direction. This behavior is attributed to the centrally symmetric boundary and loading configurations, which promote a uniform deformation response. These results demonstrate that under symmetric boundary conditions, the displacement path curves effectively capture the global deformation characteristics, reinforcing the predictive accuracy of the 2D-EPM for structural behavior prediction.

In contrast, Case 6 (CCCF) exhibits clear asymmetry in the displacement distribution due to the uneven combination of clamped (C) and free (F) boundaries. The displacement curve along Path 2 shows a pronounced gradient variation, with a significantly steeper slope near the clamped edge compared to the free edge. This nonuniform deformation arises because the clamped edge fully restricts rotation, thereby limiting displacement in that region, while the free edge permits greater deformation. As a result, the displacement profile becomes asymmetric, reflecting the influence of boundary condition imbalance on structural response.

Despite complex deformation patterns under asymmetric boundaries, the 2D-EPM accurately reproduces displacement trends, showing strong predictive capability. Across all four cases, its displacement–path curves closely match 3D-FEM results, with maximum displacement errors within acceptable engineering limits. These findings confirm the 2D-EPM’s reliability in capturing global out-of-plane deformation and boundary condition effects, providing a robust theoretical basis for structural analysis and design.

### 4.4. Out-of-Plane Bending Analysis with Initial Displacement

In practical engineering, structural components rarely possess perfect initial geometry. Sandwich panels are particularly prone to geometric imperfections introduced during manufacturing, transportation, or installation. These deviations may result from fabrication errors, material inhomogeneity, environmental influences (e.g., temperature or humidity changes), or handling disturbances. Present before service, such imperfections can markedly affect the mechanical response under out-of-plane loading–especially in thin-walled or lightweight structures–by reducing load-carrying capacity, promoting premature buckling, or inducing localized instability.

**Figure 16:**
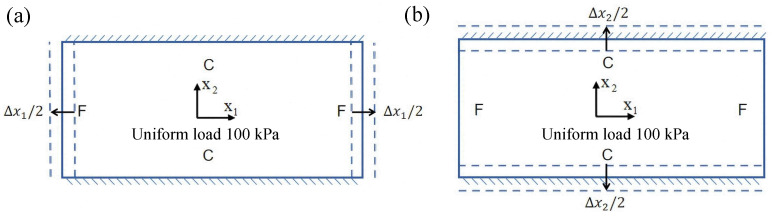
Schematic diagram of initial deformation in (**a**) $x_{1}$ and (**b**) $x_{2}$ directions.

To more accurately simulate real-world service conditions, this section incorporates initial geometric imperfections into the out-of-plane loading analysis of the SP-EHC under CCFF boundary conditions. These imperfections account for practical factors such as manufacturing deviations and residual stresses, which are commonly encountered in engineering applications. More importantly, the analysis captures the structure’s nonlinear response and potential stability issues under out-of-plane loading, providing valuable insights for the optimization and safety evaluation of sandwich panel designs. A systematic numerical investigation was conducted by introducing prescribed initial displacements of varying amplitudes into the model to evaluate their influence on the transverse load-bearing behavior and ultimate strength of the structure. The simulation procedure is illustrated in Figure 16, where $\Delta x_{1}$ and $\Delta x_{2}$ denote the imposed initial displacement amplitudes along the $x_{1}$ and $x_{2}$ directions, respectively. The range of initial imperfections considered includes −9%,−6%,−3%,0%,3%,6%, and 9%. In the FE modeling, geometric imperfections were introduced by applying an initial displacement field using the Imperfection feature in Abaqus. This approach facilitated convergence and accurately captured deformation.

**Figure 17:**
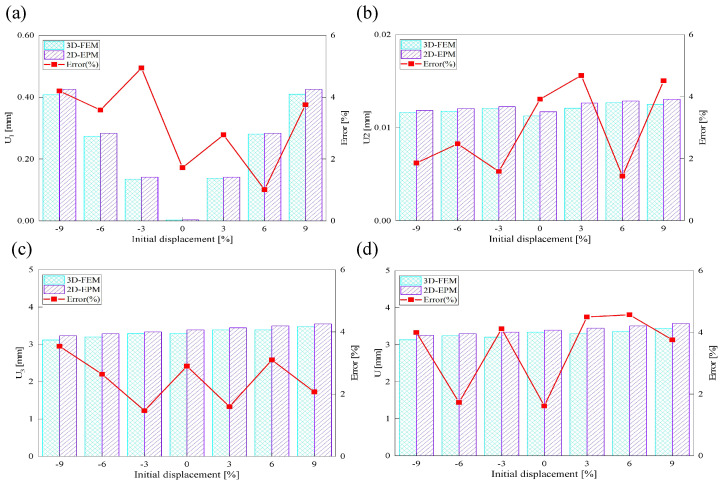
Effect of initial deformation along the $x_{1}$ direction on the displacement of SP-EHC under out-of-plane loads and CCFF boundary conditions: (**a**) $U_{1}$, (**b**) $U_{2}$, (**c**) $U_{3}$, and (**d**) *U*.

Figure 17 illustrates the influence of initial deformation along the $x_{1}$ direction on the displacement response of the SP-EHC under CCFF boundary conditions. As the initial deformation amplitude increases from −9% to 9%, the total displacement of the SP-EHC exhibits a progressive upward trend. Among the three displacement components, $U_{1}$ exhibits a nonlinear response: it first decreases and then increases with varying initial deformation. Notably, at zero imperfection (0%), $U_{1}$ is nearly zero, indicating that displacement in this direction is primarily load-induced, with minimal contribution from geometric imperfections. Furthermore, introducing moderate compressive deformation in the −$x_{1}$ direction reduces the out-of-plane displacement component $U_{3}$, effectively enhancing the panel’s out-of-plane stiffness.

**Figure 18:**
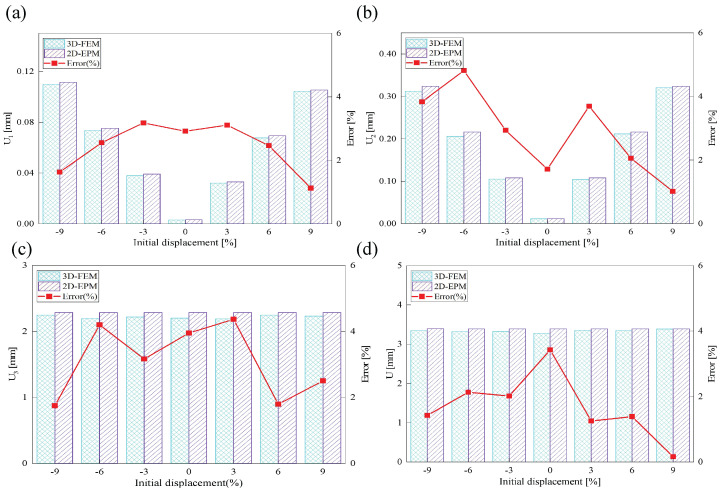
Effect of initial deformation along the $x_{2}$ direction on the displacement of SP-EHC under out-of-plane loads and CCFF boundary conditions: (**a**) $U_{1}$, (**b**) $U_{2}$, (**c**) $U_{3}$, and (**d**) *U*.

Figure 18 illustrates the influence of initial deformation along the $x_{2}$ direction on the displacement components in all three coordinate directions. As the initial deformation varies from −9% to 9%, the total displacement initially decreases and then increases, though the overall variation remains relatively small. For individual components, $U_{1}$ reaches a minimum at zero imperfection and increases with greater initial deformation, indicating a clear positive correlation. A similar trend is observed for $U_{2}$, indicating that in-plane displacements in both directions are sensitive to initial geometric imperfections. In contrast, $U_{3}$ remains relatively stable throughout the deformation range, implying that imperfections in the $x_{2}$ direction have minimal impact on the out-of-plane displacement response.

In summary, the SP-EHC under CCFF boundary conditions exhibits notable sensitivity to initial geometric imperfections. Disturbances along the $x_{1}$ and $x_{2}$ directions significantly affect the in-plane displacement components ($U_{1}$ and $U_{2}$), while their effects on the out-of-plane component ($U_{3}$) are direction-dependent. Specifically, compressive deformation along the $x_{1}$ axis contributes to a reduction in $U_{3}$, whereas deformation along the $x_{2}$ axis has negligible impact. These findings offer important guidance for controlling initial imperfections and optimizing the stability design of sandwich panels.

In summary, the 2D-EPM effectively captures in-plane stiffness and modal behavior, but it inherently overlooks out-of-plane effects like core wrinkling, face–core delamination, or transverse shear gradients. For example, in sandwich panels with complex core geometries like the SP-EHC, localized failure modes may occur under compressive or impact loads, which the 2D model cannot represent. These limitations should be considered when extending the model beyond linear elastic analysis.

### 4.5. Local Field Recovery

Local field reconstruction is an effective approach for accurately evaluating localized structural performance and informing targeted design optimizations. By enabling precise adjustments to structural configurations or material distributions, this method enhances the strength and stiffness of critical regions, thereby improving overall system reliability and stability. Compared to global response analysis, local field reconstruction offers higher spatial resolution and predictive accuracy, making it particularly well-suited for capturing localized stress, strain, and displacement fields in complex structures such as the SP-EHC.

In this section, local field reconstruction is conducted for two representative cases: (1) Case 3 without initial deformation and clamped (CCCC) boundary conditions; and (2) Case 3 with 9% initial deformation along the $x_{1}$-axis. By comparing the localized stress and displacement fields predicted by the 2D-EPM with those obtained from the 3D-FEM, the accuracy and applicability of the dimensional reduction method in reconstructing local fields are systematically evaluated.

Table 5 compares the local stress field distributions within the central cell of the panel in Case 3, as predicted by the 3D-FEM and 2D-EPM. The results indicate that the normal bending stresses ($\sigma_{11}$ and $\sigma_{22}$) are primarily concentrated in the bottom face sheet and at the face sheet–core interface, with the maximum tensile stress reaching 71.676 MPa. This stress concentration is likely caused by pronounced bending deformation in the overhanging region, driven by geometric features and external loading. The coupling between face sheet bending and core shear deformation further amplifies this localized stress.

The shear stresses ($\sigma_{13}$, $\sigma_{23}$, and $\sigma_{12}$) are primarily distributed along the cell walls of the core in the $x_{1}$-direction, with a maximum value of 18.372 MPa. Due to the high stiffness and small thickness of the face sheets, they predominantly resist bending, while the core layer bears the majority of the shear load. The observed stress heterogeneity highlights the strong coupling between load transfer and internal structure within the sandwich panel. Notably, shear stresses accumulate near the center of the face–core interface. In combination with elevated shear stresses, this significantly increases the risk of interfacial delamination. Comparison of the local stress contours reveals strong agreement between the 2D-EPM and 3D-FEM predictions, with a maximum relative error of only 1.641%. This confirms the accuracy and effectiveness of the equivalent model in capturing localized stress field behavior.

Table 6 compares the local displacement field distributions obtained from the 3D-FEM and 2D-EPM. The results reveal a pronounced displacement concentration at the face–core interface, attributed to the modulus gradient between the face sheets and the core, with the core exhibiting significantly lower stiffness. Under out-of-plane loading, the displacement distribution displays clear bi-axial symmetry, consistent with the geometric symmetry of the unit cell and the applied boundary conditions. Notably, the displacement along the $x_{1}$-direction is concentrated within the face sheet, while the displacement along the $x_{2}$-direction is concentrated near the face sheet–core interface. This behavior is attributed to the stiffness contrast between the constituent materials and the applied loading conditions. The high-stiffness face sheets dominate the deformation response along the $x_{1}$-axis, whereas the softer core material experiences greater displacement along the $x_{2}$-axis—particularly near the face–core interface—due to local stress concentrations induced by the mismatch in material stiffness.

The out-of-plane displacement component $U_{3}$ shows a red-shifted region in the contour map skewed toward the cell wall of the unit cell. This asymmetry results from stress redistribution caused by the free boundary condition in Case 3: the lack of constraint in the $x_{1}$-direction allows the cell wall to bear a larger portion of the lateral load. A quantitative comparison of the reconstructed displacement fields ($U_{1}$, $U_{2}$, $U_{3}$) shows that the average relative errors of the 2D-EPM with respect to the 3D-FEM reference are 2.7%, 1.7%, and 1.5%, respectively. These results confirm the accuracy and applicability of the proposed dimensional reduction approach in capturing the localized bending behavior of sandwich panels.

Table 7 compares the local stress fields within the central cell of the panel under the CCFF boundary condition (Case 3), with an applied initial displacement of 9%. Compared with the results in Table 6, the normal stress $\sigma_{11}$ increases by nearly an order of magnitude while $\sigma_{22}$ remains unchanged. This indicates that the imposed initial displacement in the $x_{1}$-direction significantly amplifies $\sigma_{11}$, primarily due to the lack of constraint along this direction. The resulting tensile force induced by the initial imperfection leads to elevated stress concentrations.

Moreover, high tensile stress is observed at the interface with adjacent unit cells, indicating a critical region for stress transmission. The combined effects of out-of-plane loading and initial geometric imperfection leads to stress concentration in this area. Shear stresses $\sigma_{13}$ and $\sigma_{12}$ also increase significantly—by approximately an order of magnitude—while $\sigma_{23}$ remains unchanged. This behavior may result from structural distortion near the boundary induced by the initial displacement, leading to a redistribution of internal forces. Under out-of-plane loading, the amplified local deformation intensifies both stress concentration and shear effects in this region.

Furthermore, the altered geometry introduces discontinuities in local boundary conditions, exacerbating shear stress gradients near the edges. Consequently, structural deformation and stress redistribution propagate inward from the boundaries, further intensifying the shear response. These findings highlight the importance of accounting for initial imperfections in structural core design. For cores susceptible to manufacturing or assembly defects, particular attention should be given to the strength and stiffness at key constraint regions—especially near interface boundaries and bonding lines—to mitigate stress concentrations and deformation amplification induced by initial displacements.

Table 8 compares the local displacement fields within the unit cell located at the same position under the CCFF boundary condition (Case 3) with an imposed initial displacement of 9%. By comparing these results with those in Table 6 and the corresponding displacement contours, several key observations emerge: $U_{1}$ exhibits a notable increase, $U_{2}$ shows a slight rise, and $U_{3}$ exhibits a slight reduction. This suggests that the influence of initial geometric imperfection exceeds that of out-of-plane loading, resulting in a decrease in $U_{3}$ compared to the perfect, unperturbed structure. Therefore, the impact of initial geometric imperfections must be carefully considered in the design of sandwich panels. Reinforcing or modifying regions susceptible to such defects is recommended to mitigate their adverse effects. In particular, local stiffening along directions with pronounced imperfections can help to reduce displacement concentrations and enhance overall structural robustness.

Moreover, the proposed VAM-based model has been experimentally validated in prior work. Yuan et al. [44] demonstrated its accuracy in predicting quasi-static and dynamic responses of egg-shaped honeycomb-grid sandwich panels through three-point bending and random vibration validations, confirming its suitability for complex periodic cores.

## 5. Conclusions

In this study, a novel egg-crate honeycomb core sandwich panel (SP-EHC) was proposed to address the limitations of conventional sandwich panels. A two-dimensional equivalent plate model (2D-EPM) based on the variational asymptotic method (VAM) was developed and systematically validated through experimental and finite element analyses. Based on the findings, the following conclusions can be drawn:

(1) The proposed SP-EHC exhibits superior mechanical performance, including enhanced buckling resistance and improved out-of-plane stiffness. The 2D-EPM accurately predicts both static and dynamic responses of the structure. The relative error in modal frequency prediction is within 10.23%, and the critical buckling load prediction error remains below 5.23% across various boundary conditions.

(2) The equivalent model demonstrates high fidelity in simulating bending deformations under various boundary conditions, both with and without initial geometric imperfections. Maximum displacement errors remain below 4.15%, highlighting the model’s strong applicability and reliability for practical engineering applications.

(3) The 2D-EPM effectively reconstructs local stress and displacement fields, with maximum stress prediction errors below 2.5%, demonstrating its capability in local field analysis. This offers a powerful tool for identifying stress concentrations and guiding structural optimization.

Despite the effectiveness of the VAM-based equivalent model and the novel SP-EHC structure, several limitations persist. Future work will extend the model to incorporate nonlinear material behavior, thermo-mechanical coupling, and dynamic impact response. Moreover, experimental validation under complex loading conditions, including fatigue and multi-axial stress states, is essential to evaluate the SP-EHC’s performance in practical applications.

## Figures and Tables

**Table 1:** Geometric parameters of unit cell and sandwich panels.

Model Parameter	Unit Cell						Sandwich Panel	
	$b_{1}$ (mm)	$h_{2}$ (mm)	$h_{1}$ (mm)	$t_{f}$ (mm)	$b_{2}$ (mm)	$l_{1}$ (mm)	L (mm)	W (mm)
Value	8	8	20	2	6	45	360	180

**Table 2:** Fiber/matrix/aluminum metal material parameters.

Material Parameters	Symbol/Unit	Carbon Fiber	Epoxy Resin	Aluminum
Elastic Modulus	$E_{1}$/GPa	100	4.5	70
	$E_{2}=E_{3}$/GPa	8.4	4.5	
Shear Modulus	$G_{12}=G_{13}$/GPa	4	1.7	27
	$G_{23}$/GPa	3.5	1.7	
Poisson’s Ratio	$\nu_{12}$	0.1	0.38	0.3
	$\nu_{13}$	0.25	0.38	
	$\nu_{23}$	0.25	0.38	
Density	ρ/g·cm^−3^	1.81	1.6	2.7

**Table 3:** Comparison of buckling modes and critical buckling loads under different boundaries predicted by 3D-FEM and 2D-EPM.

Case	Case 1	Case 2	Case 3	Case 4
3D-FEM	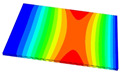	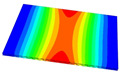	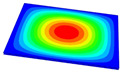	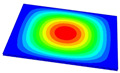
	270.63 N	533.95 N	391.00 N	411.45 N
2D-EPM	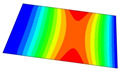	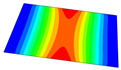	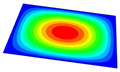	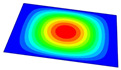
	283.05 N	551.09 N	727.10 N	745.79 N
Error	4.59%	3.21%	5.23%	2.57%

**Table 4:** Comparison of displacement clouds predicted by 3D-FEM and 2D-EPM under different boundaries (unit: mm).

Case	Case 5	Case 6	Case 7	Case 8
3D-FEM	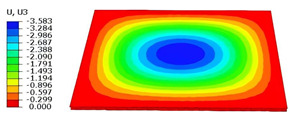	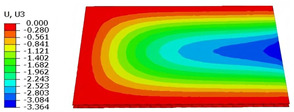	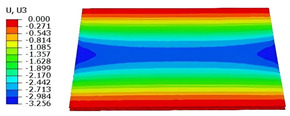	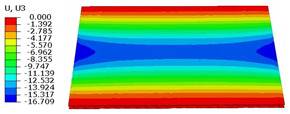
2D-EPM	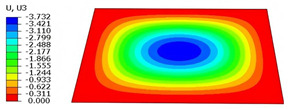	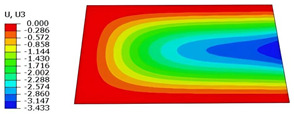	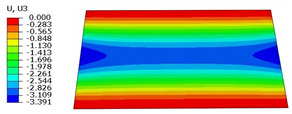	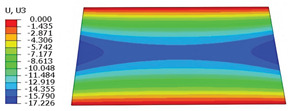
Error	2.22%	2.05%	4.15%	3.09%

**Table 5:** Comparison of local stress field distribution predicted by 3D-FEM and 2D-EPM in Case 3.

Models	$\sigma_{11}$	$\sigma_{12}$	$\sigma_{13}$
3D-FEM	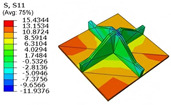	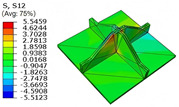	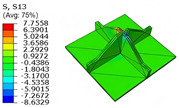
	−11.938∼15.434 MPa	−5.512∼5.546 MPa	−8.633∼7.756 MPa
2D-EPM	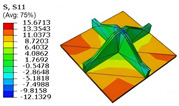	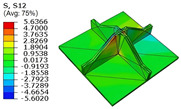	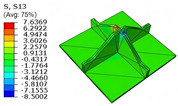
	−12.133∼15.671 MPa	−5.602∼5.637 MPa	−8.500∼7.637 MPa
Max. Error	1.536%	1.641%	1.534%
Models	$\sigma_{22}$	$\sigma_{23}$	$\sigma_{33}$
3D-FEM	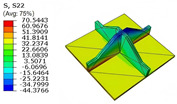	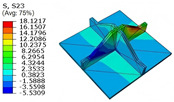	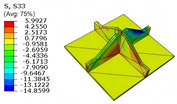
	−44.377∼70.544 MPa	−5.531∼18.122 MPa	−14.860∼5.993 MPa
2D-EPM	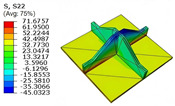	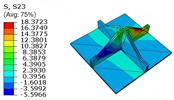	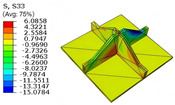
	−45.032∼71.676 MPa	−5.597∼18.372 MPa	−15.078∼6.086 MPa
Max. Error	1.610%	1.380%	1.552%

**Table 6:** Comparison of local displacement field predicted by 3D-FEM and 2D-EPM in Case 3.

Models	$U_{1}$	$U_{2}$	$U_{3}$
3D-FEM	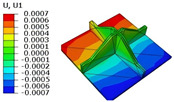	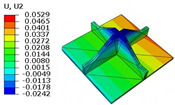	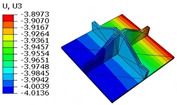
	−0.00073∼0.00074 mm	−0.0242∼0.0529 mm	−4.014∼3.897 mm
2D-EPM	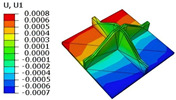	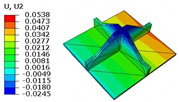	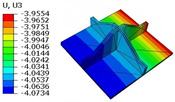
	−0.00075∼0.00076 mm	−0.0245∼0.0538 mm	−4.073∼3.955 mm
Max. Error	2.703%	1.701%	1.488%

**Table 7:** Comparison of local stress field distribution predicted by 3D-FEM and 2D-EPM in Case 3 with initial displacement of 9%.

Models	$\sigma_{11}$	$\sigma_{12}$	$\sigma_{13}$
3D-FEM	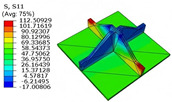	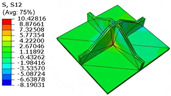	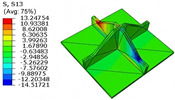
	−17.008∼112.509 MPa	−8.190∼10.428 MPa	−14.517∼13.248 MPa
2D-EPM	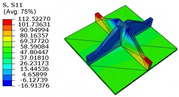	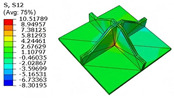	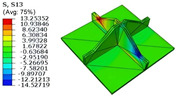
	−16.914∼112.523 MPa	−8.302∼10.518 MPa	−14.527∼13.254 MPa
Max. Error	0.552%	0.863%	0.068%
Models	$\sigma_{22}$	$\sigma_{23}$	$\sigma_{33}$
3D-FEM	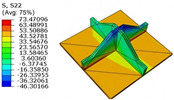	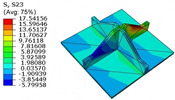	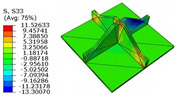
	−46.302∼73.471 MPa	−5.799∼17.542 MPa	−13.301∼11.526 MPa
2D-EPM	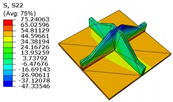	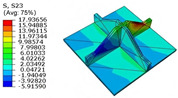	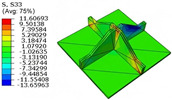
	−47.335∼75.241 MPa	−5.916∼17.937 MPa	−13.660∼11.607 MPa
Max. Error	2.409%	2.252%	0.703%

**Table 8:** Comparison of local displacement field distribution predicted by 3D-FEM and 2D-EPM in Case 3 with initial displacement of 9%.

Models	$U_{1}$	$U_{2}$	$U_{3}$
3D-FEM	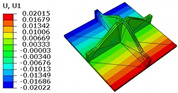	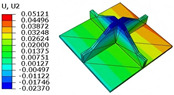	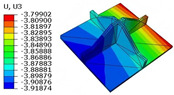
	−0.02022∼0.02015 mm	−0.0237∼0.0512 mm	−3.919∼3.799 mm
2D-EPM	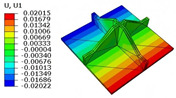	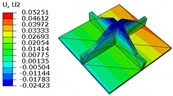	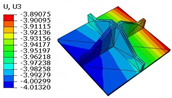
	−0.02022∼0.02015 mm	−0.0242∼0.0525 mm	−4.012∼3.891 mm
Max. Error	0.005%	2.539%	2.422%

## Data Availability

The raw data supporting the conclusions of this article will be made available by the authors on request.
